# The Foundation Fighting Blindness Plays an Essential and Expansive Role in Driving Genetic Research for Inherited Retinal Diseases

**DOI:** 10.3390/genes10070511

**Published:** 2019-07-06

**Authors:** Ben Shaberman, Todd Durham

**Affiliations:** Foundation Fighting Blindness, 7168 Columbia Gateway Drive, Suite 100, Columbia, MD 21046, USA

**Keywords:** retinitis pigmentosa, Usher syndrome, Stargardt disease, Leber congenital amaurosis, RPE65, nonprofit, patient registry, translational

## Abstract

The Foundation Fighting Blindness leads a collaborative effort among patients and families, scientists, and the commercial sector to drive the development of preventions, treatments, and cures for inherited retinal diseases (IRDs). When the nonprofit was established in 1971, it sought the knowledge and insights of leaders in the retinal research field to guide its research funding decisions. While the Foundation’s early investments focused on gaining a better understanding of the genetic causes of IRDs, its portfolio of projects would come to include some of the most innovative approaches to saving and restoring vision, including gene replacement/augmentation therapies, gene editing, RNA modulation, optogenetics, and gene-based neuroprotection. In recent years, the Foundation invested in resources such as its patient registry, natural history studies, and genetic testing program to bolster clinical development and trials for emerging genetic therapies. Though the number of clinical trials for such therapies has surged over the last decade, the Foundation remains steadfast in its commitment to funding the initiatives that hold the most potential for eradicating the entire spectrum of IRDs.

## 1. Introduction

The founders of the Foundation Fighting Blindness had no idea how challenging the development of treatments and cures for inherited retinal diseases (IRDs) would be. Little did they know, it would take nearly two decades for Foundation-funded researchers to find the first IRD gene and more than 35 years to advance a gene therapy into a human study. 

The nonprofit was established in 1971, when Eliot Berson, MD, brought together Gordon and Lulie Gund and Ben and Beverly Berman to create the first IRD research center: the Berman–Gund Laboratory for the Study of Retinal Degenerations at Massachusetts Eye and Ear Infirmary. 

At the time, Dr. Berson had recently diagnosed the Berman’s young daughters, Mindy and Joanne, with retinitis pigmentosa (RP). Gordon had recently lost all of his vision to RP after he and Lulie had completed an exhaustive search for something—anything—to save his vision. The Gund’s quest for a cure, which included a harrowing journey to a clinic in Russia at the height of the Cold War, came up empty.

It was obvious to the Foundation’s founders that virtually nothing was known about the conditions. Furthermore, they understood that no other entity—public or private—would fund research for rare retinal conditions. There was simply no commercial incentive for anyone to do so at the time. Driven by passion and a personal commitment, the small group of families took it upon themselves to get the research off the ground. Their goal was clear and singular: find preventions, treatments, and cures for everyone affected. The Berman–Gund lab was their first step forward, but little did they know how difficult the path forward would be.

“If you put your shoulder to the grindstone, we’d find an answer in five or six years,” said Lulie, reflecting on her expectations for conquering RP. “It just never occurred to me it could go on so long.”

Today, nearly 50 years later, the Foundation is the world’s largest private funding source for research to find preventions, treatments, and cures for the entire spectrum of IRDs. The nonprofit has raised more than $750 million toward its focused mission. Throughout its history, the Foundation has been led by a board and trustees comprised of families and individuals with IRDs. Likewise, it has been largely funded by grassroots donors who are also affected. Its urgent mission has been driven by those who have the greatest stake in its success.

Excitingly, there has been a tremendous surge in human research for treatments over the past 10–15 years. Nearly three dozen clinical trials for IRD therapies are underway. The US Food and Drug Administration’s (FDA’s) approval of LUXTURNA™ (Voretigene neparvovec)—the first gene therapy for the eye or an inherited condition to receive regulatory marketing approval—was a historical moment for the Foundation, which funded preclinical studies that made the sight-restoring treatment possible. The Foundation’s leadership and supporters were ebullient about the advent of the life-changing gene therapy. Finally, something made it across the finish line. Something worked, and it worked well.

However, the Foundation recognized it must optimally leverage the LUXTURNA™ approval and clinical research momentum to save the vision for the millions who still do not have any therapies. The Foundation’s funding strategy has therefore evolved from only funding basic lab research to better understand IRDs to also getting treatments across the translational chasm known as “the valley of death”—that is, to the point where biotechnology and pharmaceutical companies would invest in their clinical and commercial development. 

A little de-risking from the Foundation has gone a long way. Looking at the current IRD gene therapy and genetic treatment landscape, the Foundation’s footprint is virtually everywhere. Most current and emerging genetics-based treatments were made possible by lab, translational, and/or early clinical research funded in part by the Foundation.

In 2018, the Foundation launched its venture philanthropy fund, known as the Retinal Degeneration Fund (RD Fund), with initial capital of $70 million. Its charter is not only to fund translational and early stage clinical projects, but to attract more venture capital into the IRD space and re-invest returns back into research.

“Yes, we are a nonprofit, but that doesn’t mean we shouldn’t realize and re-invest returns for projects we are funding”, said Benjamin Yerxa, PhD, the Foundation’s chief executive officer. “The IRD gene therapy business is burgeoning, and we owe it to patients and families to leverage that momentum as much as possible to accelerate and expand therapy development.” 

While the Foundation has traditionally emphasized research to identify treatment targets and develop therapies for these genetic retinal conditions, its project portfolio has recently expanded to include natural history studies—ProgStar, for people with Stargardt disease, and RUSH2A, for those with *USH2A* mutations—as well as the global patient registry at www.MyRetinaTracker.org. An ancillary study of My Retina Tracker has thus far provided diagnostic genetic testing to approximately 4,000 IRD patients, at no cost to them. The overarching goal for these new initiatives is to gain a better understanding of how these genetic diseases affect vision, share de-identified patient data for disease progression, genetically diagnose more patients, and facilitate recruitment for clinical trials.

Data from both My Retina Tracker and the natural history studies can accelerate clinical development by helping researchers identify more powerful and sensitive clinical endpoints. 

## 2. Patient Perspectives on the Progress of Genetic Research

As mentioned, the FDA’s approval of LUXTURNA™ in December 2017 created tremendous excitement and hope for patients and families with IRDs. The success of the gene therapy program provided proof that a genetic treatment could, in fact, save and restore vision and be made commercially available to the people who need it. For the thousands of constituents affiliated with the Foundation—many of whom had been part of the organization for several decades—this was the most important and encouraging advancement in their journey. Also, the advent of additional gene therapy clinical trials in recent years—for several other IRDs, including choroideremia, X-linked RP, Stargardt disease, and Usher syndrome type 1B—boosted optimism for the potential for genetic research to halt and reverse vision loss. The Foundation’s constituents are also eager to learn about other genetic therapies, such as clustered regularly interspaced short palindromic repeats/CRISPR-associated protein 9 (CRISPR/Cas9) and antisense oligonucleotides, especially as these approaches begin to move into human studies. 

With all the enthusiasm for the current progress in research, those affected are keenly aware that only one treatment has made it through the pipeline thus far. Furthermore, LUXTURNA™ can only help a small fraction of those affected. Much more work needs to be done to address the overall need. Ultimately, sustained hope and excitement about genetic research for each patient is often predicated on the advancement of research directed toward the mutated gene causing their (or a loved one’s) IRD. 

Jen Walker, a woman with moderate vision loss from RP (*PDE6A* mutations), is excited about the LUXTURNA™ milestone, but recognizes well the unmet need and the urgency to meet it. “Hearing about LUXTURNA™ was life changing. It was astounding to see so many young people with visual impairments regain sight. The feeling of putting away a white cane for good is immeasurable,” she said, “but more work needs to be done. This is only one gene, when there are hundreds more. We need a cure quickly, as it’s going to be harder to regain sight as we lose more and more photoreceptors. I am hopeful that doctors and researchers are noticing that gene therapy for vision is an up and coming science movement, and I hope everyone gets on board sooner than later.”

John Corneille, who has advanced vision loss from RP (*PDE6B* mutations), shares Jen’s urgency for answers, but maintains an overall positive outlook. He said, “There are days, for sure, when I get discouraged thinking, at age 59, a treatment will not be found in time to enable me to see the faces of my children and grandchildren again. Most days, however, I remain very optimistic, given how far we have come in the last couple of decades. It was very exciting to learn that a company in France is engaged in a clinical trial for my gene! I try not to think about the complexity of gene studies, replacement, and editing. But it is very reassuring to know that there are countless incredibly talented researchers working hard, each day, to find breakthrough treatments for us.”

Though gene-specific therapies are often at the top of patients’ minds, more are beginning to appreciate the potential of emerging, cross-cutting genetic treatment approaches, such as optogenetics. “Perhaps most exciting is the diversity of research approaches that seem likely to eventually address any stage of these progressive and devastating diseases,” said Martha Steele, who has Usher syndrome type 2A. “As someone with advanced vision loss, I realize that not all treatments under investigation will likely work for me, but some, such as optogenetics, may well be in my future.”

Thanks to the advanced power and increasing affordability of gene-sequencing panels, more people are getting genetically tested and having their IRD gene mutation(s) identified. A genetic diagnosis can have a big impact on the patient and their family. Of course, the genetic diagnosis can put people on the path toward a clinical trial or future treatment. 

But for many patients, the identification of their gene mutation can also be cathartic. It’s a step forward in unravelling the mystery of a disease that has been progressively robbing them of their vision. For parents, the identification of their gene mutation gives them answers about the risk of passing the IRD on to their kids. Depending on the result, the knowledge can be a relief or it can raise new questions and emotions.

For Michelle Glaze, a woman with moderate vision loss from mutations in *RP1*, getting a definitive genetic diagnosis took some time, but the result helped ease her mind about her son’s risk of inheriting her IRD. “I had genetic testing done about six years ago. The initial diagnosis helped me to understand what was causing my vision loss. However, there were some missing pieces, which left some things unclear. I was not sure if my son was at risk. Thanks to additional investigation by a genetic counselor, I learned he was not at risk. Thanks to advances in genetics, and the increased ability to identify pathogenic mutations, I am now able to rest well knowing that my son will not be affected by RP. This was always a fear, always a concern in my mind, until now. As a patient and mother, I am extremely grateful for advances in research, clinical developments, and genetic testing. I have an increased hope that I may be able to see my son’s sweet face clearly one day.”

Michelle’s story underscores how critical a genetic counselor can be to the patient’s and family’s understanding and journey in managing an IRD, especially when results are inconclusive or additional testing may be advised. 

## 3. In the Beginning: The Foundation’s Early Focus on Genetics

When the Foundation began funding research in the early 1970s, one of the few clues scientists had about IRDs was that they ran in families; the conditions were clearly genetic. The nonprofit and its scientific advisors—including prominent visionaries in the retinal research community, such as John Dowling, PhD, Morton Goldberg, MD, and Alan Laties, MD—understood that identifying the genetic causes would be critical to: 1) diagnosing patients, 2) elucidating disease pathways, and 3) the development of therapies. 

As a result, throughout its early years and for decades to come, the Foundation aggressively funded (and continues to fund) the leading IRD genetic research labs around the world.

Despite its early and substantial investments in genetic research, it took nearly two decades for Foundation-funded investigators to find the first gene associated with RP (or any IRD). That gene was *RHO*, which was identified in 1989 by a team at Trinity College Dublin [1]. 

The landmark genetic breakthrough brought momentum to the search for more IRD genes, but the magnitude of the challenge was not well understood. To date, more than 270 genes have been associated with IRDs. 

“In the 1980s, we expected there would only be a handful of RP genes. We now know there are more than 80 and still counting. The effort started with a small group of scientists, across the world, working together and sharing ideas, patient samples, and lab reagents,” said Stephen Daiger, PhD, a world leader in IRD genetic research at The University of Texas Health Science Center in Houston, who has been funded by the Foundation for genetic research and discovery since 1986. “With the identification of *RHO* in 1989, the field took off. As the Human Genome Project got underway, the first useful byproduct was a much better map of human chromosomes. Because of this improved map, many more RP genes were mapped by 1995.” 

While most IRD genes have been identified, diagnostic gaps remain. Today, about two out of every three people with an IRD will have their gene mutation(s) identified when they undergo genetic diagnostic testing using a comprehensive gene panel. To address the need to genetically diagnose more patients, the Foundation is funding a five-year, $2.5 million project to find elusive IRD genes and mutations, including those in non-coding regions. The collaborative effort is being led by Dr. Daiger, Dr. Ayyagari, and Kinga Bujakowska, PhD, at Massachusetts Eye and Ear, and will include more than 140 families and an additional 400 individuals.

## 4. The Trajectory for Gene Therapy Development

With the discovery of the first genes associated with IRDs in the 1990s, the idea of developing gene replacement therapies—using viral vectors to replace mutated copies of an IRD gene with healthy copies—was tantalizingly attractive to the Foundation and its scientific advisors. After all, IRDs were caused by mutations in single genes and the retina was a clear and accessible target for such an approach. So, Foundation funding for IRD gene therapy, and relevant animal models for testing, began in earnest. 

However, for Jean Bennett, MD, PhD, and Albert Maguire, MD, the visionaries for what eventually became LUXTURNA™, the idea of gene therapy for a condition like RP came to them in medical school, well before the first IRD gene had been discovered.

“I remember in 1985, my husband, Albert Maguire, asked me if I thought we could do a gene therapy for retinitis pigmentosa. I said, sure. But what I didn’t tell him is that we didn’t know the genes, we didn’t have any animal models, and we didn’t know how to deliver DNA to the target cells,” recalled Dr. Bennett. “But that planted a seed and I started researching the state of the art. A few years later, I applied for a career development award from what was then the Retinitis Pigmentosa Foundation, now the Foundation Fighting Blindness, and got it. And that launched my whole career developing gene therapy for retinal degenerations.”

The Foundation invested approximately $10 million in *RPE65* gene therapy lab studies to enable the launch of the clinical trial in 2007 at the Children’s Hospital of Philadelphia (CHOP), which brought to fruition the vision of Drs. Bennett and Maguire. It was the first clinical trial of a gene therapy for an IRD. The company Spark Therapeutics was spun out of CHOP in 2013 to raise the money needed to get the treatment across the regulatory finish line and out to the patients who needed it. In early 2019, Spark was acquired by Roche for nearly $5 billion.

“The Foundation’s goal has been, and always will be, to get vision-saving treatments out to the people who need them. LUXTURNA™ was an important first step in achieving that goal, and we will be in business until all inherited retinal diseases are eradicated,” said Dr. Yerxa. “We are also delighted that our projects are attracting such large commercial investments, including Roche’s potential acquisition of Spark. It affirms we are on the right track with the right science, the right strategies, and the right investments.”

Several other clinical trials for IRD gene therapies were made possible by earlier Foundation funding. Take, for example, Nightstar Therapeutics’ Phase 3 clinical trial for its choroideremia gene therapy, which has preserved or improved vision for 90 percent of patients in a Phase 1/2 study. That study would not have been possible without earlier lab research by Miguel Seabra, PhD, who received more than $1.5 million from the Foundation for his efforts to characterize the *CHM* gene, develop a rodent model of choroideremia, and evaluate early versions of the *CHM* gene therapy in lab studies. Nightstar was recently acquired by Biogen for approximately $800 million.

Large animal models and related safety and efficacy studies have been invaluable to the advancement of IRD gene therapies, and perhaps no other Foundation-funded lab has been more productive in IRD large animal research than the University of Pennsylvania School of Veterinary Medicine. Its successful studies in canines have led to gene therapy clinical trials for: Leber congenital amaurosis (*RPE65* mutations), X-linked RP (*RPGR* mutations), and achromatopsia (*CNGA3* and *CNGB3* mutations). Human trials resulting from its Best disease and RP (*RHO* mutations) gene therapy canine studies are currently being planned. 

## 5. Beyond Gene Replacement

While momentum for the clinical development of gene replacement therapies for IRDs is strong, the approach has its limitations. 

For example, the cargo capacity of the adeno-associated viruses (AAVs) commonly (and successfully) used for gene delivery in LUXTURNA™ and most ongoing clinical trials is limited to about 4.7 kb. Several genes, including *ABCA4* (Stargardt disease), *USH2A* (RP and Usher syndrome), and *CEP290* (LCA) exceed the AAV’s capacity.

Also, for autosomal dominant IRDs, such as RP caused by mutations in *RHO*, the delivery of a replacement gene will not be sufficient; a therapy will need to silence the mutated allele encoding the toxic protein or the allele acting in a dominant-negative fashion. 

In recent years, the Foundation’s research portfolio has expanded to include gene-editing treatment approaches such as CRISPR/Cas9 for autosomal dominant RP caused by mutations in *RHO* (Johns Hopkins and Columbia) and RP1 (Massachusetts Eye and Ear), as well as Usher syndrome type 1B caused by mutations in *MYO7A* (UCLA). 

In February 2018, the Foundation Fighting Blindness, through its RD Fund, announced funding of up to $7.5 million for the development of ProQR’s QR-421a, an antisense oligonucleotide (AON) designed to block mutations in RNA caused by defects in exon 13 of *USH2A*. ProQR announced in March 2019 that it had dosed the first patient in its Phase 1/2 clinical trial for QR-421a. Excitingly, the company reported vision improvements for 60 percent of participants in its Phase 1/2 targeting a recurrent mutation in *CEP290*, which causes LCA10. A Phase 2/3 trial for the LCA10 AON is now underway.

## 6. Cross-Cutting Gene Therapies

Even before the first gene replacement therapy clinical trial got off the ground (the RPE65 trial at CHOP) in 2007, Foundation-funded scientists were envisioning neuroprotective gene-therapy paradigms that could help people regardless of the mutated gene causing their disease. That is, delivering a gene to express proteins that would slow photoreceptor degeneration. 

Neuroprotection became attractive to Foundation leadership and scientific advisors because of the technical and financial infeasibility of developing a gene replacement therapy for the hundreds of mutated genes that cause IRDs. According to RetNet (https://sph.uth.edu/retnet/) there are more than 270 genes associated with IRDs. Furthermore, approximately one third of patients will not have their mutation(s) identified when genetically tested. 

In 2005, José Sahel, MD, and Thierry Léveillard, PhD, at the Institut de la Vision, received the Foundation’s Board of Director’s Award for identifying a protein produced and secreted by rod photoreceptors that prevented cones from degenerating in models of RP. Aptly named the rod-derived cone-viability factor (RdCVF), the protein was an intriguing approach for saving cone-mediated vision in people with RP and related conditions. Perhaps most appealing was that RdCVF had the potential to work independent of the patient’s mutated gene—an approach that would be desirable for those whose gene mutation could not be identified, or those for whom gene replacement or editing wasn’t technically desirable. 

The newly-formed French company SparingVision plans to advance RdCVF into a clinical trial soon, thanks to the culmination of many years of lab funding from the Foundation and its recent commitment of up to €7 million.

The Foundation is also funding optogenetic therapies—the delivery of a gene to retinal ganglion or bipolar cells to express a light-sensitive protein in a retina that has lost all its photoreceptors due to an advanced IRD. In fact, the Foundation funded preclinical research for retinal optogenetic approaches currently in clinical trials—studies sponsored by Allergan and GenSight. The Foundation is also funding John Flannery, PhD, UC Berkeley, who is developing optogenetic alternatives designed to work in more natural lighting conditions. 

While still in early clinical trials, optogenetic therapies hold promise for restoring meaningful vision to people who have lost all of their photoreceptors, regardless of the mutated gene causing their blindness.

## 7. Natural History Studies: Learning about Disease Progression and Genotype–Phenotype Correlations 

The successful development of any new therapy requires a thorough understanding of the disease—in the absence of treatment—ideally from the time of diagnosis to its end stages. Understanding this natural history of disease enables clinical researchers to describe the clinical manifestations of disease (the phenotype) and its association with the genotype, estimate how quickly the disease progresses over time, identify patient characteristics that predict slower or faster disease progression, and study which clinical assessments are most appropriate to measure a treatment’s benefit. Addressing these objectives is particularly important for IRDs because they are highly variable in their clinical manifestations, they may progress over decades, and because they are rare diseases about which little may be known. Ultimately, the knowledge gained from natural history studies will provide a number of key insights. This fundamental work will inform the designs of clinical trials of new treatments, the patient population most likely to benefit, the length of follow-up required to demonstrate a benefit, and the outcomes that are most sensitive to change [2].

The Foundation funds and conducts natural history studies of IRDs through its Clinical Consortium, a coordinating center and an international group of over 25 leading research centers which are experts in IRDs. The Clinical Consortium’s mission is to accelerate the development of treatments for IRDs through collaborative and transparent clinical research. These objectives are met by ensuring the studies are designed, led, and reported by participating investigators and by making the study datasets publicly available for wider use. Because the studies are conducted using industry standards for quality—including good clinical practice (GCP) and site certification for retinal imaging modalities—the Foundation has designed the studies so the data will have broad utility, including, in some situations, to serve as a historical control. 

Currently, the Foundation’s Clinical Consortium is conducting RUSH2A, a prospective, four-year, natural history study of approximately 100 patients with an IRD associated with mutations in the *USH2A* gene, the most common mutated gene in Usher syndrome type 2 and a frequent cause of non-syndromic RP. The primary objectives of the RUSH2A study are to characterize the progression of the disease with respect to functional outcome measures (e.g., visual acuity and static perimetry) and structural outcome measures (e.g., the area of the ellipsoid zone measured by SD-OCT), to investigate the relationships between structure and function, and to assess whether there are genotypic or phenotypic predictors of progression at four years.

By the end of 2019, the Clinical Consortium plans to initiate a natural history study of retinal dystrophy associated with the *EYS* gene, PRO-EYS. The PRO-EYS study has similar objectives to RUSH2A and will follow approximately 100 patients for four years. A key feature of PRO-EYS is that the patient population will be stratified by the severity of disease at study entry. Thus, the study will provide valuable information that can be used to design trials for treatments at various stages of disease progression. 

Natural history studies of IRDs and their associated pathogenic genes will continue to be a major activity of the Foundation’s Clinical Consortium. These studies have broad applicability; they therefore represent an ideal partnership opportunity for industry sponsors, who can save time and effort by leveraging the network’s existing research infrastructure and access to IRD patients around the world.

## 8. My Retina Tracker: The Foundation’s Global Patient Registry

Patient data for IRDs—both genetic and phenotypic information—is rare. Furthermore, IRD patient data collected by academic research centers is usually not shared widely and often limited to the conditions studied by the institution. 

However, the need for comprehensive IRD patient data has become paramount with the surge in clinical trials for emerging therapies. The success of these human studies depends greatly on a sponsor’s ability to recruit enough genotypically and phenotypically well-characterized patients.

In 2014, the Foundation launched its secure global patient registry, My Retina Tracker (www.MyRetinaTracker.org), to provide pre-screened researchers and companies with de-identified patient and disease data for relevant studies, including IRD clinical trials and natural history studies.

The registry is patient controlled; the patient uploads and maintains their own record. When a company or researcher searches the registry for potential clinical trial participants, they never receive patient names or personal information. Instead, they are sent an alphanumeric identifier, which Foundation administrators use to identify and notify the patient who matched the search criteria. It is then up to the patient to contact the clinical trial coordinator about possible participation in the trial or study. 

As of June 2019, more than 12,260 patients (with an informative profile of their disease) were registered in My Retina Tracker. Approximately 400 new patients register every month.

The Foundation Fighting Blindness has been conducting a genetic testing study for patients registered in My Retina Tracker. Through the study, registrants obtain genetic testing, at no cost to them. A 266 IRD gene panel (includes copy number variation testing) is being used to screen DNA samples. No cost genetic counseling is provided for those patients who don’t receive genetic counseling from their clinic or physician.

“Genetically characterizing IRD patients and making their de-identified molecular and disease information available to the research community is critical for advancing human disease and therapy studies,” said Brian Mansfield, PhD, the Foundation’s executive vice president research and interim chief scientific officer. “Dozens of therapy developers and investigators from around the world have used data from My Retina Tracker to advance their lab and clinical research. With approximately 200,000 IRD patients in the United States alone, we still have a lot of work to do, but we are building momentum as more patients learn about My Retina Tracker and the genetic testing study.” 

## 9. Conclusion: Filling the Gaps to Advance the Field 

The Foundation’s role in driving genetic research for IRDs has evolved and expanded as a result of advancement in biological sciences, the development of powerful gene sequencing technologies, the mapping of the human genome, and the growth in its own revenues and membership base. Of course, success in gene therapy development—including the regulatory approval of LUXTURNA™ and the impressive results from preclinical research that propelled the *RPE65* gene therapy toward the clinic—has also brought accelerating momentum to clinical development in the field.

However, the Foundation has always maintained (and continues to maintain) a commitment to funding projects that would fill critical gaps in research that were not addressed by commercial or government sectors, especially when doing so advanced the entire IRD field. 

Today, the My Retina Tracker patient registry, genetic testing study, and Stargardt disease and *USH2A* natural history studies are all prime examples of significant Foundation investments that are having a wide-reaching impact in the advancement of research, especially when it comes to the clinical development of sight-saving and -restoring therapies. In most cases, these are major investments, each costing several millions of dollars, which other organizations haven’t been able or willing to make.

The Foundation’s long-standing, guiding imperative—whatever the investment—is to ensure that it is based on good science and it will get more preventions, treatments, and cures for IRDs across the finish line for everyone affected.
